# Circadian Regulation of Neuronal Membrane Capacitance—Mechanisms and Implications for Neural Computation and Behavior

**DOI:** 10.3390/ijms262110766

**Published:** 2025-11-05

**Authors:** Agnieszka Nowacka, Maciej Śniegocki, Dominika Bożiłow, Ewa Ziółkowska

**Affiliations:** 1 Department of Neurosurgery, Nicolaus Copernicus University in Toruń, Collegium Medicum in Bydgoszcz, ul. Curie Skłodowskiej 9, 85-094 Bydgoszcz, Poland; sniegocki@cm.umk.pl; 2 Anaesthesiology and Intensive Care Clinical Ward, The 10th Military Research Hospital and Polyclinic, ul. Powstańców Warszawy 5, 85-681 Bydgoszcz, Poland; bozilow@wp.pl; 3 Department of Pediatrics, School of Medicine, Washington University in St. Louis, St. Louis, MO 63110, USA

**Keywords:** circadian rhythms, membrane capacitance, neuronal excitability, synaptic integration, neural computation, chronotherapy, neurodegeneration, biophysics

## Abstract

Neuronal membrane capacitance (Cm) has traditionally been viewed as a static biophysical property determined solely by the geometric and dielectric characteristics of the lipid bilayer. Recent discoveries have fundamentally challenged this perspective, revealing that Cm exhibits robust circadian oscillations that profoundly influence neural computation and behavior. These rhythmic fluctuations in membrane capacitance are orchestrated by intrinsic cellular clocks through coordinated regulation of molecular processes including transcriptional control of membrane proteins, lipid metabolism, ion channel trafficking, and glial-mediated extracellular matrix remodeling. The dynamic modulation of Cm directly impacts the membrane time constant (τm = RmCm), thereby altering synaptic integration windows, action potential dynamics, and network synchronization across the 24 h cycle. At the computational level, circadian Cm oscillations enable neurons to shift between temporal summation and coincidence detection modes, optimizing information processing according to behavioral demands throughout the day–night cycle. These biophysical rhythms influence critical aspects of cognition including memory consolidation, attention, working memory, and sensory processing. Disruptions in normal Cm rhythmicity are increasingly implicated in neuropsychiatric and neurodegenerative disorders, including depression, schizophrenia, Alzheimer’s disease, and epilepsy, where altered membrane dynamics compromise neural circuit stability and information transfer. The integration of circadian biophysics with chronomedicine offers promising therapeutic avenues, including chronotherapeutic strategies that target membrane properties, personalized interventions based on individual chronotypes, and environmental modifications that restore healthy biophysical rhythms. This review synthesizes evidence from molecular chronobiology, cellular electrophysiology, and systems neuroscience to establish circadian Cm regulation as a fundamental mechanism linking molecular timekeeping to neural computation and behavior.

## 1. Introduction

The conceptual framework underlying our understanding of neuronal membrane capacitance (Cm) has undergone a revolutionary transformation over the past decade [1]. Historically, membrane capacitance was considered a fixed biophysical parameter, determined solely by the geometric properties and dielectric characteristics of the neuronal lipid bilayer, with the relationship Cm = εA/d (where ε represents the dielectric constant, A the membrane area, and d the bilayer thickness) [2]. This static view positioned capacitance as an unchanging cellular property that, while fundamental to neural computation, remained constant throughout a neuron’s functional lifespan [3]. However, mounting experimental evidence has shattered this traditional paradigm, revealing that neuronal membrane capacitance exhibits pronounced rhythmic fluctuations across the circadian cycle [4,5].

The recognition of dynamic capacitance regulation represents a paradigm shift with profound implications for neuroscience [6]. Membrane capacitance directly determines the membrane time constant through the relationship τm = RmCm, where Rm represents membrane resistance, fundamentally governing how neurons integrate synaptic inputs and generate action potentials [7,8]. The central hypothesis underlying this review is that rhythmic modulation of membrane capacitance serves as a fundamental mechanism by which circadian clocks dynamically adjust neuronal computational properties to optimize information processing according to time-of-day-dependent behavioral demands. When Cm oscillates rhythmically, it introduces temporal variation in neuronal excitability, synaptic integration windows, and spike timing precision, effectively modulating the computational strategies available to individual neurons and neural networks throughout the day–night cycle [9,10]. This dynamic regulation enables neurons to adaptively adjust their information processing capabilities according to behavioral demands and environmental contexts that vary across circadian time [11].

The discovery of circadian oscillations in membrane biophysical properties emerged from convergent lines of evidence spanning molecular chronobiology, cellular electrophysiology, and systems neuroscience [12,13]. Pioneering work by Severín et al. provided direct electrophysiological evidence that cortical pyramidal neurons and hippocampal granule cells exhibit statistically significant daily fluctuations in membrane capacitance, with amplitudes reaching 15–20% of baseline values [1]. These findings built upon earlier studies demonstrating that core clock genes, including Clock, Bmal1, Per, and Cry, are expressed not only in canonical circadian pacemaker neurons but throughout the central nervous system, where they regulate the rhythmic expression of genes encoding membrane proteins, lipid enzymes, and ion channels [14,15]. Subsequent electrophysiological investigations revealed that these molecular oscillations translate into measurable changes in neuronal membrane properties, including capacitance, with amplitudes sufficient to significantly alter neural computation [16,17]. The integration of these findings has established circadian regulation of membrane capacitance as a fundamental mechanism linking molecular timekeeping to neural function [18].

The functional significance of capacitance rhythmicity extends far beyond individual cellular properties to encompass network dynamics and behavioral outputs [19,20]. Oscillations in Cm modulate the membrane time constant, thereby influencing the temporal window during which synaptic inputs can be integrated and the precision with which action potentials are generated [21]. These changes propagate through neural circuits to affect network oscillations, information transfer efficiency, and ultimately cognitive performance across domains including memory consolidation, attention, working memory, and sensory processing [22,23]. The temporal structure imposed by circadian Cm regulation ensures that neural circuits operate optimally within the environmental framework of the 24 h day, maximizing performance during periods of heightened behavioral demand while conserving resources during rest phases [24].

Clinical investigations have increasingly implicated disruptions in circadian membrane biophysics in the pathogenesis of diverse neuropsychiatric and neurodegenerative disorders [25,26]. Conditions ranging from depression and bipolar disorder to Alzheimer’s disease and epilepsy exhibit characteristic disturbances in sleep–wake cycles, cognitive rhythms, and neural excitability that may reflect underlying abnormalities in capacitance regulation [27,28]. The recognition that membrane capacitance oscillations serve as a critical interface between molecular clocks and higher-order brain function has opened new therapeutic avenues through chronomedicine approaches that target the temporal organization of neural systems [29,30].

The translational potential of understanding circadian capacitance regulation is substantial, encompassing pharmacological interventions timed to coincide with optimal membrane states, personalized medicine approaches based on individual chronotypes, and environmental modifications that restore healthy biophysical rhythms [31,32]. These chronotherapeutic strategies promise to improve treatment efficacy while minimizing side effects by aligning interventions with the intrinsic temporal structure of neural function [33]. Furthermore, the development of biomarkers based on membrane biophysical rhythms could enable early detection of circadian dysregulation and facilitate preventive interventions before the onset of overt pathology [34].

This comprehensive review synthesizes current knowledge regarding the mechanisms underlying circadian regulation of neuronal membrane capacitance and examines its implications for neural computation, behavior, and disease. We explore the molecular and cellular processes that generate capacitance rhythms, analyze their computational and network-level consequences, and discuss their relevance to cognition, behavior, and clinical disorders. Finally, we consider translational perspectives and future directions for integrating circadian biophysics with chronomedicine to advance both fundamental understanding and therapeutic applications.

## 2. Circadian Systems and Neuronal Biophysics

The hierarchical organization of circadian timing systems provides the temporal architecture within which neuronal membrane capacitance oscillations are coordinated and regulated [35]. Understanding this organizational framework is essential for appreciating how molecular clockwork mechanisms translate into the dynamic modulation of membrane biophysical properties across neural circuits and brain regions [36]. The mammalian circadian system operates through a multi-tiered network comprising central pacemaker neurons in the suprachiasmatic nucleus (SCN) and distributed peripheral oscillators throughout the nervous system, each contributing distinct yet coordinated roles in temporal regulation [37].

### 2.1. Hierarchical Organization of Circadian Clocks

The suprachiasmatic nucleus serves as the master circadian pacemaker, orchestrating behavioral and physiological rhythms through its intrinsic capacity to generate self-sustained oscillations in neuronal activity [38]. Individual SCN neurons exhibit autonomous rhythmicity in electrical firing patterns, maintaining stable circadian periodicity in vitro and in tissue culture preparations, demonstrating their robust cell-autonomous clock properties [39]. This intrinsic oscillatory capacity emerges from the molecular transcription-translation feedback loops that operate within each SCN neuron, where core clock proteins including CLOCK, BMAL1, PERIOD (PER1-3), and CRYPTOCHROME (CRY1-2) interact to generate approximately 24 h rhythms in gene expression [40,41].

The molecular architecture underlying SCN function involves positive and negative regulatory elements that create stable oscillations through delayed feedback inhibition [42]. CLOCK and BMAL1 heterodimerize to form transcriptional activators that bind to E-box elements in the promoters of Per and Cry genes, driving their rhythmic expression during the subjective day [43]. The resulting PER and CRY proteins accumulate, form complexes, and translocate to the nucleus where they inhibit CLOCK-BMAL1-mediated transcription, creating the negative limb of the feedback loop [44]. The temporal delay introduced by protein synthesis, post-translational modifications, nuclear entry, and subsequent proteolytic degradation establishes the approximately 24 h periodicity characteristic of circadian rhythms [45].

Beyond the SCN, peripheral circadian oscillators are distributed throughout the brain and peripheral tissues, each possessing the same core molecular clockwork but exhibiting tissue-specific patterns of gene expression and functional output [46,47]. These peripheral clocks are present in neurons, astrocytes, microglia, and other cell types within the central nervous system, where they regulate local aspects of cellular physiology including membrane properties [48]. Importantly, peripheral oscillators maintain their rhythmicity when isolated from the SCN, demonstrating their cell-autonomous nature, but require periodic synchronization signals from the master pacemaker to maintain coherent phase relationships across tissues [49,50].

The coordination between central and peripheral clocks occurs through multiple signaling pathways including direct neural projections, circulating hormones, and metabolic cues [51]. The SCN projects to hypothalamic and extra-hypothalamic brain regions through well-defined neural circuits that convey timing information via neurotransmitters and neuropeptides including vasopressin, vasoactive intestinal peptide, and GABA [52]. These signals entrain peripheral oscillators by influencing their molecular clockwork, ensuring that local cellular rhythms remain synchronized with the environmental light-dark cycle [53].

### 2.2. Integration with Neuronal Function

The integration of circadian timing systems with neuronal biophysical properties represents a fundamental mechanism by which temporal information is encoded at the cellular level [54]. Clock gene expression within individual neurons directly influences the transcriptional regulation of genes encoding membrane proteins, ion channels, and enzymes involved in lipid metabolism, creating the molecular foundation for rhythmic changes in membrane capacitance [55,56]. This transcriptional control extends beyond the core clock machinery to encompass approximately 10–15% of the genome in a tissue-specific manner, including numerous genes that determine neuronal excitability and membrane properties [57].

The coupling between molecular oscillators and membrane biophysics occurs through multiple mechanisms operating at different temporal scales [58]. At the transcriptional level, CLOCK-BMAL1 complexes directly regulate the expression of ion channels including voltage-gated sodium, potassium, and calcium channels whose density and distribution influence membrane capacitance [59,60]. Post-translational modifications add an additional layer of regulation, with rhythmic phosphorylation, acetylation, and other modifications altering channel gating properties, trafficking, and membrane insertion throughout the circadian cycle [61,62].

Cell-type-specific circadian regulation emerges from the differential expression of clock-controlled genes across neuronal populations [63]. Excitatory pyramidal neurons and inhibitory interneurons exhibit distinct patterns of circadian gene expression, reflecting their specialized functional roles within neural circuits [64]. For example, parvalbumin-positive fast-spiking interneurons show particularly robust rhythms in genes encoding extracellular matrix components and membrane-associated proteins, correlating with their unique biophysical properties and high metabolic demands [65,66]. These cell-type differences in molecular clock outputs likely contribute to the observed heterogeneity in membrane capacitance oscillations across neuronal populations.

The functional consequences of clock–membrane integration extend to activity-dependent processes that shape neural circuit development and plasticity [67]. Circadian regulation of membrane properties influences the timing and magnitude of calcium influx during synaptic activity, thereby affecting gene expression programs involved in synaptic strengthening and homeostatic scaling [68,69]. This creates a bidirectional relationship where molecular clocks influence membrane excitability, while activity-dependent signaling feeds back to modulate clock gene expression and phase relationships [70].

Environmental entrainment pathways provide additional complexity to clock–membrane interactions [71]. Light information detected by specialized retinal ganglion cells is transmitted to the SCN via the retinohypothalamic tract, where it influences core clock gene expression and subsequently affects downstream targets including membrane-related genes [72,73]. Temperature cycles, feeding rhythms, and social cues represent non-photic zeitgebers that can also influence peripheral clocks and their membrane-related outputs through distinct signaling mechanisms [74,75].

**Figure 1:**
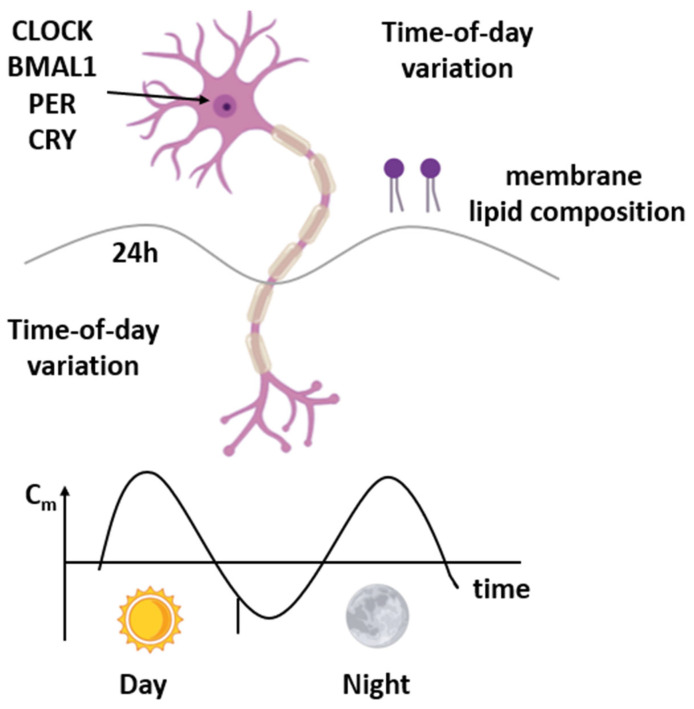
Conceptual overview of circadian regulation of neuronal membrane capacitance (Cm)—this schematic illustration shows how molecular clock components (CLOCK, BMAL1, PER, and CRY) within the neuronal nucleus coordinate daily changes in membrane lipid composition and ion channel properties. These oscillations lead to time-of-day variations in membrane capacitance (Cm), which increase during the active (day) phase and decrease during the rest (night) phase, reflecting rhythmic regulation of neuronal excitability.

The temporal coordination of membrane capacitance oscillations across different brain regions requires precise communication between central and peripheral clocks [76]. Disruptions in this coordination, whether through genetic mutations affecting core clock components, environmental perturbations such as shift work or jet lag, or pathological processes, can lead to internal desynchronization and aberrant membrane dynamics [77,78]. Such dysregulation has been implicated in various neuropsychiatric and neurodegenerative disorders where altered membrane properties contribute to circuit dysfunction and behavioral abnormalities [79,80]. The integration of molecular circadian mechanisms with biophysical membrane dynamics is summarized conceptually in Figure 1.

## 3. Biophysical Foundations and Measurement

The quantitative characterization of neuronal membrane capacitance requires a comprehensive understanding of its physical determinants, measurement methodologies, and the technical challenges associated with detecting circadian oscillations in this fundamental biophysical parameter [3]. Modern electrophysiological approaches have evolved considerably to address the complexities introduced by neuronal morphology, membrane heterogeneity, and the dynamic nature of capacitance regulation, providing increasingly sophisticated tools for investigating circadian modulation of membrane properties [81].

### 3.1. Membrane Capacitance: Definition and Determinants

Membrane capacitance represents the ability of the neuronal plasma membrane to store electrical charge, arising from the lipid bilayer’s function as a biological capacitor separating conductive intracellular and extracellular compartments [82]. The specific capacitance per unit area depends fundamentally on the dielectric properties of the membrane material and its thickness, with typical values for biological membranes ranging from 0.8 to 1.2 μF/cm^2^ [83,84]. Recent precise measurements using advanced patch-clamp techniques have revealed that mammalian neurons exhibit specific membrane capacitance values closer to 0.9 μF/cm^2^, with remarkably consistent values across different neuronal cell types despite significant variations in membrane protein content [85].

The total membrane capacitance of a neuron reflects the integration of specific capacitance across the entire cell surface, making it directly proportional to membrane surface area [86]. This relationship has profound implications for understanding circadian capacitance oscillations, as changes in total Cm could arise from either alterations in specific membrane properties or modifications in effective surface area through processes such as membrane trafficking, spine dynamics, or extracellular matrix remodeling [1,87]. The distinction between these mechanisms is crucial for interpreting experimental observations and understanding the molecular basis of capacitance rhythmicity [88].

The membrane time constant, defined as τm = RmCm where Rm represents input resistance, emerges as the critical parameter linking biophysical properties to neuronal computation [7]. This relationship demonstrates how circadian oscillations in Cm directly translate into temporal changes in synaptic integration windows, action potential dynamics, and network synchronization properties [8]. Recent theoretical and experimental work has emphasized that even modest changes in Cm, on the order of 10–20%, can produce functionally significant alterations in neuronal responsiveness and information processing capabilities [89,90]. Specifically, a 15% increase in Cm prolongs τm proportionally, extending the temporal window for synaptic integration from milliseconds to tens of milliseconds, thereby shifting neurons from coincidence detection toward temporal integration modes [1]. Conversely, reduced Cm during other circadian phases shortens integration windows, enhancing precision in spike timing and responsiveness to synchronous inputs.

### 3.2. Structural and Molecular Contributors

The molecular architecture underlying membrane capacitance encompasses multiple interacting components that contribute to both baseline values and their circadian modulation [91]. The lipid bilayer itself, composed primarily of phospholipids, cholesterol, and specialized membrane lipids, provides the fundamental dielectric properties that determine specific capacitance [92]. Circadian regulation of lipid metabolism, including rhythmic expression of enzymes involved in phospholipid synthesis and fatty acid desaturation, represents a key mechanism by which molecular clocks could influence membrane electrical properties [15,29].

Ion channels and membrane proteins, while contributing minimally to total capacitance under normal conditions, can indirectly influence effective membrane capacitance through their effects on membrane structure and local electrical properties [93,94]. The rhythmic expression and trafficking of voltage-gated channels, neurotransmitter receptors, and membrane transporters under circadian control provides additional pathways for temporal modulation of membrane biophysical properties [4,5]. Post-translational modifications of these proteins, including phosphorylation, glycosylation, and lipidation, add further layers of circadian regulation that can fine-tune membrane capacitance throughout the day–night cycle [62].

Perineuronal nets (PNNs) have emerged as critical modulators of neuronal membrane capacitance, particularly in fast-spiking parvalbumin-positive interneurons where they enable sustained high-frequency firing [22,27,95,96,97]. These specialized extracellular matrix structures, composed primarily of chondroitin sulfate proteoglycans, hyaluronic acid, and link proteins, form condensed lattice-like networks around neuronal cell bodies and proximal dendrites [22,95,96,98]. Recent investigations have demonstrated that PNNs significantly decrease specific membrane capacitance by increasing effective membrane thickness and altering local dielectric properties [22,96,99,100].

The dynamic regulation of PNN composition and density exhibits robust circadian rhythmicity across multiple brain regions, with diurnal fluctuations in proteoglycan content, enzymatic remodeling activity, and structural integrity [22,101]. Matrix metalloproteinases and other PNN-degrading enzymes show time-of-day-dependent activity patterns that could contribute to circadian oscillations in membrane capacitance by rhythmically modifying the extracellular matrix environment surrounding neurons [102]. This mechanism represents a novel pathway linking circadian clocks to membrane biophysics through extracellular matrix remodeling rather than direct intracellular modifications [103].

### 3.3. Methodological Considerations

The accurate measurement of neuronal membrane capacitance presents significant technical challenges, particularly when attempting to detect circadian oscillations that may exhibit relatively small amplitude changes against background variability [3]. Current-clamp protocols, involving hyperpolarizing or depolarizing current steps and analysis of the resulting voltage responses, have emerged as the gold standard for capacitance measurement in morphologically complex neurons [104,105]. These approaches avoid artifacts associated with voltage-clamp measurements in non-isopotential cells and provide more reliable estimates of total membrane capacitance [106].

The exponential fitting of voltage responses to current steps requires careful attention to series resistance compensation, membrane leak correction, and the presence of voltage-dependent conductance that can contaminate capacitance measurements [107,108]. Recent methodological advances have emphasized the importance of using multiple exponential components to accurately capture the complex voltage dynamics in neurons with extensive dendritic arbors, where spatial heterogeneity in membrane properties can produce multi-phasic responses [109,110].

Cell-type specificity represents a crucial consideration in studies of circadian capacitance regulation, as different neuronal populations exhibit distinct baseline capacitance values, morphological complexity, and patterns of clock gene expression [23]. Excitatory pyramidal neurons and inhibitory interneurons show markedly different capacitance characteristics, with fast-spiking parvalbumin-positive interneurons exhibiting particularly pronounced effects of PNN-mediated capacitance modulation [111,112]. The identification and characterization of these cell-type differences are essential for understanding the functional significance of capacitance oscillations in neural circuit operation [21].

Regional variability in membrane properties adds another layer of complexity to capacitance measurements, with neurons in different brain areas exhibiting distinct developmental trajectories, morphological features, and susceptibility to circadian modulation [113,114]. Cortical layer-specific differences in capacitance values and rhythmicity patterns reflect underlying variations in cellular architecture, connectivity patterns, and local circuit demands [115,116]. Recent high-throughput electrophysiological studies have begun to map these regional and laminar differences systematically, providing foundational data for understanding the systems-level organization of capacitance regulation [117,118].

The temporal resolution required for detecting circadian oscillations necessitates experimental protocols that can capture capacitance dynamics across multiple time points while controlling for confounding factors such as temperature fluctuations, pH changes, and metabolic state variations [16,17]. Advanced automated patch-clamp systems and long-term recording approaches are beginning to enable the continuous monitoring of membrane properties over extended periods, opening new possibilities for characterizing the precise temporal dynamics of capacitance oscillations [119,120].

Despite these methodological advances, several challenges persist in the reliable measurement of membrane capacitance (Cm). Small fluctuations in series resistance, seal quality, and electrode capacitance can introduce significant errors, particularly during long-term recordings needed to assess circadian changes. The non-isopotential geometry of neurons, especially those with extensive dendritic trees, leads to spatial filtering that distorts Cm estimation and complicates the interpretation of multi-exponential fits. Temperature and pH variations can alter membrane dielectric properties, introducing artifactual oscillations in measured capacitance values. Moreover, biological noise, phototoxicity during simultaneous optical monitoring, and drift in patch stability over 24 h cycles further constrain the precision and reproducibility of Cm measurements. Overcoming these limitations will require improved long-term recording stability, refined analytical models accounting for neuronal morphology, and standardized procedures for environmental control across circadian experiments [16,17,104,106,108].

## 4. Mechanisms of Circadian Capacitance Regulation

The rhythmic modulation of neuronal membrane capacitance emerges from coordinated molecular and cellular processes operating at multiple scales, from transcriptional regulation of membrane-associated genes to structural remodeling of neuronal membranes and their extracellular environment [28]. Understanding these mechanisms is crucial for appreciating how circadian clocks translate molecular oscillations into functionally relevant changes in neuronal biophysical properties that influence computation and behavior [36].

### 4.1. Evidence for Oscillatory Dynamics

Direct electrophysiological measurements have provided compelling evidence for circadian oscillations in neuronal membrane capacitance across multiple species and brain regions [5]. The landmark discovery by Severin et al. demonstrated that cortical pyramidal neurons and hippocampal granule cells exhibit statistically significant daily fluctuations in membrane capacitance, with amplitudes reaching 15–20% of baseline values [1]. These oscillations closely track circadian time, peaking during specific phases of the light-dark cycle in a manner consistent with endogenous clock regulation rather than indirect metabolic or activity-dependent effects [4].

The temporal dynamics of capacitance oscillations reveal cell-type specificity, with excitatory principal neurons showing robust rhythmicity while certain inhibitory interneuron populations, notably parvalbumin-positive fast-spiking cells, exhibit distinct patterns or absent oscillations [23]. This differential regulation suggests that circadian control of membrane properties is tailored to the functional requirements of specific neuronal populations within neural circuits [112]. Regional heterogeneity further characterizes capacitance rhythms, with neurons in different brain areas displaying varied amplitude, phase, and sensitivity to environmental perturbations [57].

The relationship between membrane capacitance and membrane time constant oscillations provides functional validation of these biophysical changes [3]. Measurements demonstrate that circadian fluctuations in Cm produce corresponding alterations in τm, with periods of high capacitance associated with prolonged membrane time constants that favor temporal summation, while low capacitance phases facilitate rapid membrane charging and coincidence detection [7]. These temporal changes in integration properties correlate with behavioral state transitions and cognitive performance patterns across the circadian cycle [11].

Cell-type-specific variability in capacitance rhythms reflects intrinsic differences in neuronal morphology, membrane composition, and clock gene expression. Larger neurons with extensive dendritic arbors, such as pyramidal cells, tend to exhibit greater oscillatory amplitudes due to higher membrane surface area and dynamic regulation of ion channel distribution. In contrast, compact interneurons with limited dendritic complexity display attenuated or irregular rhythms, potentially reflecting divergent coupling between intrinsic circadian mechanisms and membrane remodeling pathways. Such variability underscores the importance of considering neuronal identity and electrotonic architecture when interpreting capacitance oscillations in relation to circadian function [21,23,111].

### 4.2. Molecular Mechanisms

The molecular and cellular mechanisms underlying circadian Cm regulation operate through coordinated pathways spanning transcriptional control, metabolic regulation, membrane trafficking, and extracellular matrix dynamics. These mechanisms converge to produce coherent oscillations in membrane electrical properties that scale from individual neurons to network-level function. Understanding these mechanisms requires integration of insights from molecular biology, lipid biochemistry, cellular trafficking, and glial biology.

#### 4.2.1. Transcriptional Control and Gene Expression

The molecular clockwork exerts transcriptional control over membrane capacitance through rhythmic regulation of genes encoding membrane lipid enzymes, ion channels, and structural proteins [6]. CLOCK-BMAL1 heterodimers bind to E-box elements in the promoters of numerous clock-controlled genes whose products directly or indirectly influence membrane properties [40,43]. Chromatin immunoprecipitation studies have identified >150 direct CLOCK-BMAL1 target genes in neurons, including key enzymes in phospholipid biosynthesis (e.g., CDP-choline pathway enzymes showing 2–3 fold circadian amplitude) and ion channel genes exhibiting robust daily expression rhythms [54,56]. Comprehensive transcriptomic analyses have identified extensive circadian regulation of genes involved in phospholipid biosynthesis, sphingolipid metabolism, and cholesterol homeostasis, all of which contribute to membrane biophysical characteristics [55,56].

Recent investigations have revealed that sphingolipid metabolism represents a critical pathway linking circadian clocks to membrane remodeling [121]. In Drosophila circadian circuits, specific sphingolipids exhibit robust diurnal fluctuations in the brain, with glial control of glucocerebrosidase activity sculpting the lipid environment surrounding clock neurons [122]. Disruption of sphingolipid homeostasis through genetic manipulation of gba1b produces alterations in neuronal structure and circadian behavior, demonstrating the functional significance of lipid metabolic rhythms for neural circuit organization [123].

The transcriptional architecture underlying lipid metabolic control involves multiple regulatory layers beyond core clock components [15]. Nuclear receptors including REV-ERBα and RORα, which form secondary feedback loops within the molecular clock, directly regulate genes encoding rate-limiting enzymes in lipid biosynthetic pathways [124,125]. This integration of metabolic and temporal information ensures that membrane remodeling processes are coordinated with cellular energy status and nutritional availability [126].

Ion channel genes represent another major class of clock-controlled transcripts that influence membrane capacitance through their effects on channel density and distribution [127]. Voltage-gated sodium, potassium, and calcium channels show rhythmic expression patterns in suprachiasmatic nucleus neurons and other circadian-relevant brain regions [128,129]. The circadian regulation of channel transcription provides a mechanism for time-of-day-dependent modulation of neuronal excitability that complements direct capacitance effects [130].

#### 4.2.2. Lipid Metabolism and Membrane Remodeling

Circadian rhythms in brain lipid metabolism orchestrate dynamic remodeling of neuronal membranes, directly impacting membrane capacitance through alterations in bilayer composition, thickness, and dielectric properties [95,96,131]. The brain exhibits particularly robust circadian regulation of lipid homeostasis, reflecting the high metabolic demands of neural tissue and the critical importance of membrane integrity for neuronal function [132,133]. Recent lipidomic studies have revealed extensive daily fluctuations in phospholipid species, with specific molecular forms of phosphatidylcholine, phosphatidylethanolamine, and phosphatidylserine showing peak abundance at distinct circadian phases [70].

The enzymatic machinery controlling membrane lipid synthesis displays pronounced circadian rhythmicity in expression and activity [134]. Fatty acid synthase, acetyl-CoA carboxylase, and other key enzymes in de novo lipogenesis exhibit time-of-day-dependent regulation that generates oscillations in lipid production rates [135]. Similarly, phospholipid biosynthetic enzymes including CDP-choline pathway components show circadian expression patterns that coordinate with cellular demands for membrane expansion or remodeling [136].

Membrane remodeling processes involve coordinated regulation of lipid synthesis, trafficking, and degradation pathways [137]. Phospholipases that catalyze membrane phospholipid hydrolysis show circadian activity patterns, generating lysophospholipids and fatty acids that serve as signaling molecules and substrates for membrane restructuring [138]. The balance between anabolic and catabolic lipid pathways under circadian control determines net changes in membrane composition and potentially surface area, both factors that influence total membrane capacitance [139].

The emerging field of circadian lipidomics has revealed that different brain regions exhibit distinct lipid metabolic rhythms, reflecting local neural activity patterns and circuit-specific demands [140]. Recent work has demonstrated that circadian disruption through genetic clock mutations or environmental perturbations produces profound alterations in brain lipid profiles associated with impaired cognitive function and increased vulnerability to neurodegenerative processes [141,142]. These findings underscore the clinical relevance of maintaining proper temporal coordination of lipid metabolic pathways for neural health [131].

#### 4.2.3. Ion Channel Distribution and Trafficking

Dynamic regulation of ion channel trafficking and membrane insertion represents a rapid mechanism for modulating membrane capacitance and neuronal excitability across circadian cycles [127,143]. Ion channels undergo continuous cycles of internalization and reinsertion at the plasma membrane through endocytic and exocytic pathways that can be regulated by circadian signals [144]. Recent evidence demonstrates that the density and spatial distribution of voltage-gated channels exhibit time-of-day-dependent variations that influence both membrane electrical properties and action potential dynamics [145,146].

The molecular machinery controlling channel trafficking shows circadian regulation at multiple levels [147]. Small GTPases including Rab proteins that coordinate vesicular transport exhibit rhythmic expression and activity patterns [148]. SNARE proteins mediating membrane fusion events during channel insertion also display circadian modulation, providing a mechanism for temporal control over channel surface expression [149]. Post-translational modifications including phosphorylation, ubiquitination, and palmitoylation influence channel trafficking rates and show circadian rhythmicity through clock-regulated kinase and ligase activities [150,151].

Specific ion channel families demonstrate distinct trafficking patterns across the circadian cycle [152]. Voltage-gated potassium channels including Kv2.1 undergo activity-dependent redistribution that appears modulated by circadian factors, with clustering patterns and phosphorylation states varying with time of day [153]. Sodium channels responsible for action potential initiation show circadian variations in axon initial segment density that correlate with changes in neuronal excitability [154]. Calcium channels exhibit complex trafficking regulation involving circadian modulation of auxiliary subunits that influence channel stability at the membrane [155].

The functional consequences of rhythmic channel trafficking extend beyond simple changes in channel number to encompass alterations in channel composition and properties that fine-tune membrane responses [15]. Circadian regulation of channel auxiliary subunits can modify gating kinetics, voltage dependence, and pharmacological sensitivity, effectively generating time-of-day-specific channel phenotypes [156,157]. These dynamic adjustments in channel properties complement capacitance changes to optimize neuronal computation for different behavioral contexts across the circadian cycle [158]. The major ion channel types subject to circadian regulation and their impact on neuronal excitability and capacitance are summarized in Table 1.

#### 4.2.4. Glial Cell Contributions and Myelin Dynamics

Glial cells play essential roles in regulating neuronal membrane capacitance through their control of the extracellular environment, myelin dynamics, and metabolic support [48,159]. Oligodendrocytes, the myelinating glia of the central nervous system, exhibit intrinsic circadian rhythms in gene expression and metabolic activity that influence the structural and electrical properties of the myelin sheaths they produce [160,161]. Recent investigations have revealed that myelin plasticity, including changes in sheath thickness and compaction, occurs across developmental and experiential timescales, with emerging evidence suggesting circadian modulation of these processes [162,163,164].

The impact of myelination on neuronal capacitance arises from the addition of multiple membrane wraps around axons, effectively increasing membrane thickness and decreasing specific capacitance per unit axonal length [87]. Theoretical modeling demonstrates that even modest changes in myelin thickness or paranodal sealing can significantly alter effective membrane capacitance and conduction velocity [165]. The optimization of myelin geometry represents an evolutionary balance between maximizing signal transmission speed while minimizing metabolic costs associated with maintaining extensive membrane structures [166].

Astrocytes contribute to capacitance regulation through their modulation of extracellular space geometry and ionic composition [167,168]. These cells exhibit robust circadian rhythms in metabolic activity, gene expression, and morphological properties [169]. Astrocytic processes that ensheath synapses and neuronal somata undergo dynamic remodeling that varies with sleep–wake cycles and circadian time, potentially influencing local electrical field properties and effective neuronal capacitance [170,171].

**Figure 2:**
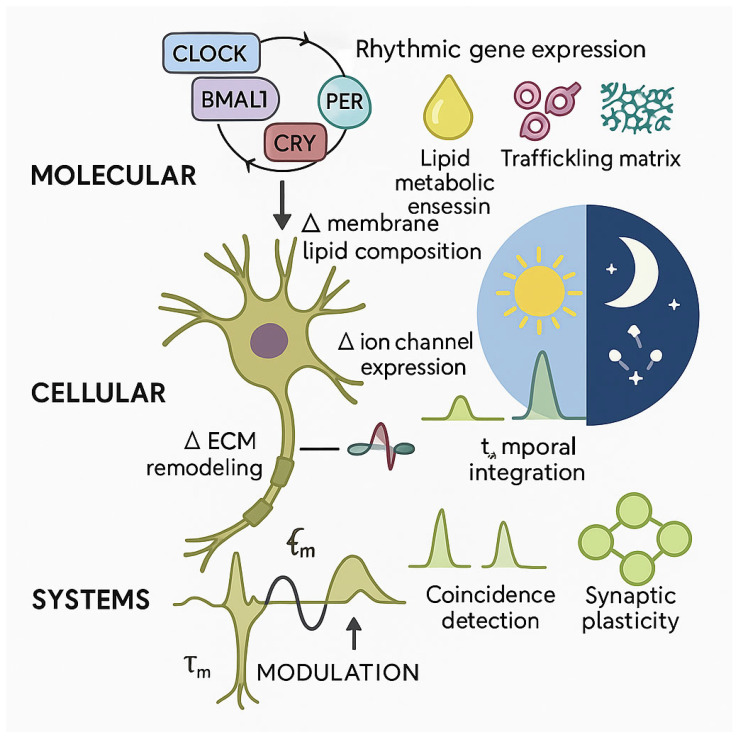
Multi-scale integration of circadian mechanisms regulating neuronal membrane capacitance—the mechanisms regulating circadian Cm oscillations operate across multiple biological scales. At the molecular level, the core clock machinery (CLOCK-BMAL1-PER-CRY feedback loops) drives rhythmic transcription of genes encoding lipid metabolic enzymes, ion channels, and trafficking machinery. At the cellular level, these molecular rhythms translate into coordinated changes in membrane lipid composition, ion channel surface expression, and glial-mediated extracellular matrix remodeling. These cellular changes converge to produce measurable oscillations in membrane capacitance that directly modulate the membrane time constant (τm). At the systems level, altered τm shifts neuronal computational modes between temporal integration and coincidence detection, affecting network synchronization, synaptic plasticity rules, and ultimately cognitive performance across the day–night cycle. This multi-scale integration establishes circadian Cm regulation as a fundamental link between molecular clocks and behavioral outputs.

The synthesis and remodeling of perineuronal nets represents a particularly important glial contribution to capacitance regulation [98]. Astrocytes and specialized extracellular matrix-secreting cells produce the chondroitin sulfate proteoglycans, hyaluronan, and link proteins that comprise PNN structures [172]. Recent work has demonstrated that PNN assembly and disassembly show circadian rhythmicity, with matrix metalloproteinase activity varying across the day–night cycle to sculpt PNN density and composition [22,103]. This dynamic regulation provides a mechanism for adjusting neuronal capacitance through changes in the electrical properties of the neuronal microenvironment rather than intrinsic membrane modifications [27]. The principal regulatory levels contributing to circadian oscillations of neuronal membrane capacitance are summarized in Table 2 and Figure 2.

### 4.3. Developmental, Aging, and Sex Differences in Capacitance Oscillations

The expression and regulation of membrane capacitance rhythms undergo substantial modifications across the lifespan, reflecting changing demands on neural circuits during development, maturation, and aging [173,174]. Developmental trajectories of capacitance oscillations correlate with the establishment of mature circadian rhythms and the refinement of neural circuit connectivity [175]. In early postnatal development, neuronal membrane properties exhibit high plasticity, with capacitance values changing substantially as dendrites elaborate and synaptic connections form [90,176].

The emergence of robust circadian capacitance oscillations parallels the maturation of molecular clock function in developing neurons [177]. Neonatal neurons initially show weak or absent membrane property rhythms despite expressing core clock genes, suggesting that additional maturational processes are required for establishing functional coupling between molecular oscillators and membrane biophysics [178]. Critical period plasticity windows may be particularly sensitive to disruptions in emerging capacitance rhythms, with implications for neurodevelopmental disorders characterized by altered circadian function [103,179].

Aging processes profoundly impact both baseline membrane capacitance values and the amplitude of circadian oscillations [77,173]. Older neurons typically exhibit increased input capacitance associated with age-related changes in dendritic complexity and membrane composition [109]. Importantly, the robustness of circadian capacitance rhythms declines with advanced age, paralleling deterioration in other circadian outputs including sleep–wake cycles, hormone secretion, and cognitive performance [180,181]. This age-related dampening of biophysical rhythms may contribute to increased vulnerability to neurological disorders in elderly populations [79].

Sex differences in neuronal membrane properties and their circadian regulation represent an emerging area of investigation with implications for understanding sex-specific vulnerabilities to psychiatric and neurological disorders [182,183]. Gonadal hormones including estrogens and androgens influence membrane lipid composition, ion channel expression, and synaptic connectivity through mechanisms that interact with circadian clock function [184,185]. Female rodents exhibit distinct patterns of capacitance regulation compared to males, with estrous cycle stage modulating the amplitude and phase of circadian oscillations in membrane properties [186,187].

The mechanisms underlying sex differences in capacitance rhythms involve both organizational effects during development and activational effects in adulthood [188]. Sex chromosomes contribute directly through genes expressed from the X and Y chromosomes that influence neuronal differentiation and function [189]. Additionally, sex differences in circadian clock gene expression and phase relationships between central and peripheral oscillators may underlie differential regulation of membrane properties in males versus females [190,191].

## 5. Computational and Network Consequences

The circadian modulation of neuronal membrane capacitance fundamentally alters how individual neurons and neural networks process information, with cascading effects on computation from subcellular integration to systems-level dynamics [10]. These biophysical oscillations introduce temporal structure into neural computation that must be considered when analyzing information processing, synaptic plasticity, and network function across the day–night cycle [192].

### 5.1. Single-Cell Computation

#### 5.1.1. Membrane Time Constant Modulation

The membrane time constant τm = RmCm serves as the fundamental temporal filter governing neuronal responsiveness to synaptic inputs [193]. Circadian oscillations in membrane capacitance directly modulate this time constant, shifting the temporal integration window available for summing incoming signals [7]. When Cm increases during certain circadian phases, the prolonged τm extends the period over which subthreshold synaptic potentials can accumulate, enhancing sensitivity to temporally distributed inputs and favoring integration modes of computation [16,194].

Conversely, reductions in Cm during other circadian phases shorten τm, enabling neurons to respond more rapidly to synaptic inputs with reduced temporal summation [81]. This shortened integration window enhances coincidence detection capabilities, allowing neurons to selectively respond to synchronous inputs while filtering out temporally dispersed signals [195]. The dynamic adjustment of temporal filtering properties enables individual neurons to adopt computational strategies appropriate for different behavioral contexts across the circadian cycle [196].

The relationship between membrane capacitance and temporal filtering has been demonstrated experimentally through precise electrophysiological measurements showing that even modest changes in Cm produce functionally significant alterations in voltage response kinetics [3]. Recent computational modeling confirms that circadian-scale variations in capacitance can shift neurons between integrator and coincidence detector modes, fundamentally altering their input-output transformations [1,197].

#### 5.1.2. Action Potential Dynamics

Circadian oscillations in membrane capacitance profoundly influence action potential generation and propagation through effects on voltage dynamics and threshold characteristics [198]. The rate of membrane depolarization during synaptic integration depends critically on the membrane time constant, with higher Cm values slowing voltage changes and potentially elevating action potential threshold [89,90]. These alterations in spike initiation dynamics can modify neuronal gain, changing the relationship between input current and firing rate across circadian time [199].

Spike timing precision represents another critical parameter affected by capacitance oscillations [200]. Neurons with lower Cm exhibit reduced temporal jitter in action potential generation, enabling more precise encoding of rapid input fluctuations [201]. This enhanced temporal fidelity may be particularly important during circadian phases demanding high-precision sensory processing or motor coordination [202]. Conversely, periods of elevated Cm may favor robustness over precision, promoting stable firing patterns that are less sensitive to brief perturbations [203].

Axonal conduction velocity shows sensitivity to membrane capacitance through its influence on the speed of action potential propagation along axons [87]. Lower specific membrane capacitance facilitates faster conduction by reducing the time required to charge membrane segments ahead of the propagating spike [85]. Recent work has demonstrated that reductions in Cm, whether through myelin optimization or other mechanisms, can increase conduction velocity by substantial margins with significant implications for network synchrony and information transfer speed [165,166].

The backpropagation of action potentials into dendritic arbors, crucial for coincidence detection and synaptic plasticity induction, depends critically on dendritic membrane properties including capacitance [8]. Circadian modulation of Cm may alter the reliability and extent of backpropagating action potentials, thereby influencing the spatial and temporal requirements for inducing long-term synaptic modifications [21,204].

**Figure 3:**
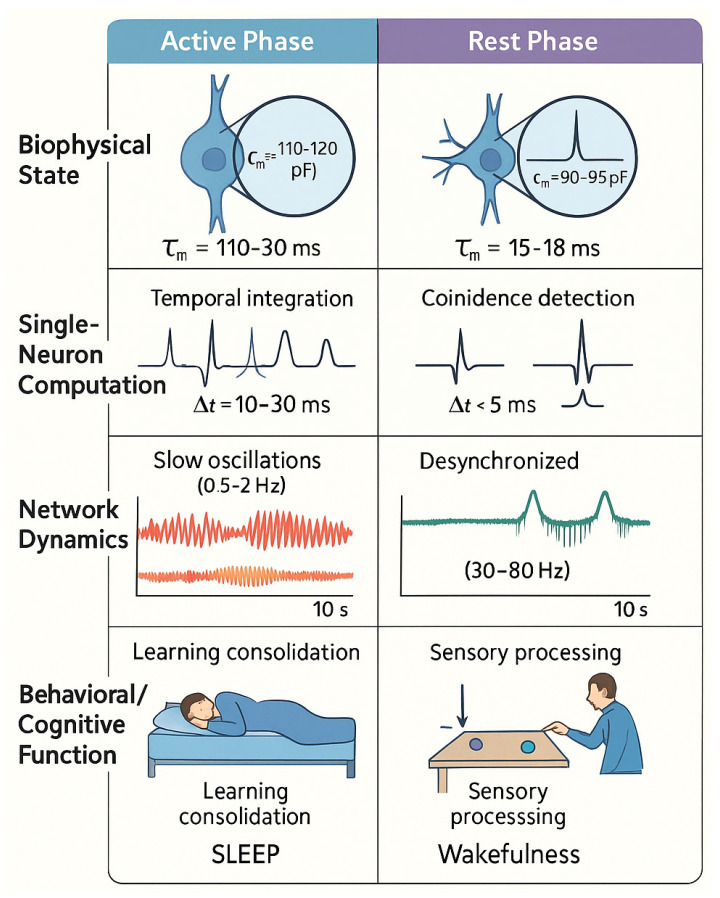
Circadian modulation of neuronal computational modes and network dynamics—circadian oscillations in membrane capacitance shift neurons between computational modes. Active phase (left): High Cm (110–120 pF) and prolonged τm (25–30 ms) favor temporal integration, where EPSPs separated by 10–30 ms summate effectively. Networks exhibit slow oscillations and sustained synchronization, optimizing memory consolidation during sleep. Rest phase (right): Low Cm (90–95 pF) and shortened τm (15–18 ms) favor coincidence detection, requiring synchronous inputs (Δt < 5 ms). Networks exhibit theta and gamma oscillations with transient synchronization, enhancing sensory precision and attention. Bottom panel shows resulting circadian variations in cognitive performance across domains. Gradual transitions (2–3 h) allow adaptive tuning while maintaining stability. Abbreviations: Cm, membrane capacitance; τm, membrane time constant; EPSP, excitatory postsynaptic potential; ZT, zeitgeber time.

Figure 3 illustrates the computational consequences of circadian Cm modulation through side-by-side comparison of high Cm (temporal integration) and low Cm (coincidence detection) states.

### 5.2. Synaptic Plasticity and Learning

Spike-timing-dependent plasticity (STDP), the canonical mechanism linking precise temporal patterns of pre- and postsynaptic activity to synaptic strength modifications, operates within temporal windows determined by membrane dynamics [33,205]. The millisecond-scale precision required for STDP induction depends on membrane time constants that govern both postsynaptic potential kinetics and backpropagating action potential timing [206]. Circadian oscillations in membrane capacitance therefore modulate the temporal windows and efficacy of STDP by altering these fundamental biophysical parameters [34].

Recent experimental evidence has revealed that STDP rules exhibit time-of-day-dependent variations, with different circadian phases showing distinct requirements for the relative timing of pre- and postsynaptic spikes to induce potentiation or depression [23,69]. These temporal variations in plasticity rules likely reflect underlying changes in membrane capacitance and associated time constants that shift the temporal integration properties critical for detecting spike coincidences [207]. However, it is important to note that these studies have been conducted primarily in rodent models and specific brain regions (visual cortex, hippocampus). The generalizability across species, brain regions, and cell types remains an active area of investigation. Furthermore, the relative contributions of Cm changes versus concurrent alterations in ion channel expression and neuromodulator tone to STDP variability require systematic dissection through targeted experimental manipulations.

The relationship between STDP and behavioral learning timescales has long presented a conceptual challenge, as millisecond-precision spike timing must somehow support learning processes occurring over seconds to hours [208]. Circadian modulation of membrane capacitance may provide a bridge across these temporal scales by creating distinct epochs during which specific plasticity rules are favored, effectively gating learning processes according to behavioral state and time of day [67,70].

Metaplasticity, the plasticity of synaptic plasticity itself, represents another layer at which circadian capacitance oscillations influence learning mechanisms [209]. By modulating baseline neuronal excitability and integration properties, capacitance rhythms establish distinct metaplastic states that determine the threshold and direction of subsequent synaptic modifications [210,211]. This temporal structuring of metaplasticity may optimize learning by coordinating plasticity induction with periods of heightened attention or memory consolidation [212].

Critical periods in development, characterized by heightened neural plasticity and circuit refinement, exhibit particular sensitivity to disruptions in circadian regulation [179,213]. Emerging evidence suggests that proper circadian modulation of membrane capacitance may be necessary for establishing normal critical period boundaries and ensuring appropriate circuit maturation [103]. Disturbances in these developmental processes could contribute to neurodevelopmental disorders characterized by both circadian dysfunction and aberrant neural connectivity [214].

### 5.3. Network-Level Effects

Network oscillations, fundamental to diverse cognitive processes including attention, memory, and sensory processing, depend critically on the temporal coordination of neuronal activity [9,215]. Membrane capacitance influences network dynamics through its effects on single-neuron excitability, synaptic integration, and spike timing, all of which contribute to the generation and maintenance of synchronized activity patterns [216]. Circadian modulation of Cm therefore introduces temporal structure into network oscillations, potentially optimizing different oscillatory regimes for distinct behavioral demands across the day–night cycle [217].

The synchronization of neuronal ensembles requires precise temporal alignment of action potentials across distributed populations [218]. Changes in membrane capacitance alter the speed and reliability of synaptic integration and spike generation, thereby influencing the ease with which neurons can synchronize their activity [17,219]. Periods of low Cm may favor high-frequency oscillations requiring rapid, precise spike timing, while elevated Cm during other phases could promote slower, more robust oscillatory patterns less dependent on millisecond-scale precision [220].

Functional connectivity between brain regions emerges from coordinated patterns of neural activity that facilitate information transfer [221]. The communication-through-coherence hypothesis proposes that effective information transmission occurs when sending and receiving neural populations oscillate in phase, creating temporal windows of heightened receptivity [192,222]. Circadian modulation of membrane capacitance could influence these phase relationships by altering the temporal response properties of neurons in different brain regions, potentially optimizing connectivity patterns for different cognitive demands at different times of day [223].

Signal-to-noise ratio, critical for reliable information processing, depends on the balance between signal-carrying neural responses and background activity [224]. Membrane capacitance influences this balance through its effects on temporal filtering and neuronal gain [225]. Lower Cm may enhance signal-to-noise ratio by improving temporal precision and reducing integration of uncorrelated background activity, while higher Cm could favor signal detection in noisy environments by increasing temporal summation of weak signals [226,227].

Excitation–inhibition balance represents a fundamental organizing principle in neural circuits, with disruptions implicated in numerous neuropsychiatric conditions [228,229]. Recent findings demonstrate that excitation–inhibition balance itself exhibits circadian rhythmicity, with daily oscillations in the relative strength of excitatory and inhibitory inputs [23]. Given that inhibitory interneurons, particularly fast-spiking parvalbumin-positive cells, show distinct patterns of membrane capacitance regulation compared to excitatory neurons, circadian modulation of Cm may contribute to these daily fluctuations in circuit balance [27,112].

## 6. Behavioral and Cognitive Implications

The circadian modulation of neuronal membrane capacitance exerts profound influences on cognitive performance and behavior through its effects on neural information processing, synaptic plasticity, and network dynamics [19,20]. These biophysical rhythms provide a mechanistic foundation for understanding the well-documented time-of-day variations in cognitive abilities, sensory processing, and motor performance observed across species [11].

### 6.1. Memory Formation and Consolidation

Memory consolidation, the process by which newly acquired information is stabilized and integrated into long-term storage, exhibits robust circadian regulation that appears intimately linked to neuronal membrane dynamics [67,230]. Sleep-dependent memory consolidation relies critically on coordinated patterns of neural activity including slow oscillations, sleep spindles, and hippocampal sharp-wave ripples, all of which depend on precise temporal coordination of neuronal firing determined in part by membrane time constants [231,232].

The coupling between slow oscillations and spindles during non-rapid eye movement (NREM) sleep plays a central role in memory consolidation [233]. Recent evidence demonstrates that the temporal precision of this coupling correlates with memory performance, suggesting that circadian modulation of membrane capacitance could influence consolidation efficacy by altering the ease with which neurons synchronize during these critical oscillatory states [234,235]. Membrane capacitance values directly affect the temporal integration windows available for detecting coincident synaptic inputs during memory reactivation events, potentially gating which memories undergo successful consolidation [236].

The synaptic homeostasis hypothesis posits that sleep serves to downscale synaptic strengths that have been potentiated during waking, preventing saturation and maintaining network stability [237,238]. Circadian oscillations in membrane capacitance may contribute to this process by modulating neuronal excitability and integration properties in a manner that favors either synaptic strengthening during wake or depotentiation during sleep [239]. The temporal structure imposed by capacitance rhythms could thus coordinate molecular, synaptic, and network-level processes underlying memory consolidation [70].

Recent work has revealed circadian disruption impairs memory consolidation even when total sleep duration remains unchanged, suggesting that proper temporal alignment of biophysical properties is as important as sleep itself [240,241,242]. In Drosophila models, disruption of circadian clock function in specific neuronal populations produces memory deficits that correlate with altered synaptic plasticity and network activity patterns [243]. These findings underscore the essential role of intact circadian regulation of membrane properties for optimal cognitive function [244].

### 6.2. Working Memory and Executive Functions

Working memory, the cognitive system responsible for temporarily holding and manipulating information, shows pronounced circadian variation in capacity and efficiency [245,246]. Executive functions including inhibitory control, cognitive flexibility, task switching, and decision-making exhibit time-of-day effects that reflect underlying changes in prefrontal cortical network dynamics [247,248]. These cognitive domains are particularly sensitive to circadian phase and show optimal performance when testing time aligns with an individual’s chronotype preference [249,250].

The neural substrates of working memory depend critically on sustained neuronal activity patterns that maintain information representations over delay periods [251]. The stability and precision of these activity patterns rely on membrane properties including capacitance that determine temporal integration and firing reliability [252]. Circadian modulation of Cm could influence working memory capacity by altering the number of stable activity states that neuronal networks can maintain simultaneously [253].

Executive functions show greater circadian sensitivity compared to more automated cognitive processes, with tasks requiring inhibition of prepotent responses or flexible adaptation to changing rules exhibiting particularly pronounced time-of-day effects [254,255]. This differential sensitivity may reflect the metabolic demands and network complexity underlying executive control, making these functions more vulnerable to suboptimal biophysical states [256]. Recent studies demonstrate that executive function performance correlates with circadian markers including core body temperature and melatonin rhythms, supporting a mechanistic link between physiological oscillations and cognitive capacity [247,257,258,259].

Age-related changes in working memory and executive function parallel deterioration in circadian rhythm amplitude and phase stability [180,181]. Older adults exhibit reduced time-of-day effects on some cognitive tasks, potentially reflecting dampened oscillations in membrane capacitance and other circadian outputs [260]. The preservation of robust circadian rhythms through lifestyle interventions may therefore represent a strategy for maintaining cognitive function during aging [77,79].

### 6.3. Attention, Filtering, and Cognitive Flexibility

Attentional systems responsible for selecting relevant information from competing sensory inputs show circadian modulation that influences both sustained attention and selective filtering capabilities [261,262]. The temporal dynamics of attentional orienting and engagement depend on neural circuit properties including membrane time constants that govern response latencies and integration windows [263]. Circadian oscillations in Cm could therefore modulate attentional performance by altering the temporal characteristics of information processing in sensory and association cortices [264].

Cognitive flexibility, the ability to adaptively shift strategies or mental sets in response to changing demands, requires dynamic reconfiguration of network connectivity patterns [265,266]. This reconfiguration depends on the balance between network stability and plasticity, which is influenced by intrinsic membrane properties [267]. Circadian regulation of capacitance may facilitate appropriate shifts between stable and flexible network states across the day–night cycle, supporting cognitive flexibility when behavioral demands require adaptation [268].

The filtering of irrelevant distractors represents a critical component of attentional control that shows time-of-day variation [269,270]. Inhibitory mechanisms responsible for suppressing distractors depend on precise temporal coordination within prefrontal-parietal networks, coordination that could be influenced by circadian modulation of membrane dynamics [271]. Individual differences in circadian preference correlate with distinct patterns of attentional control, with morning-types and evening-types showing optimal filtering performance at different times of day [272,273].

Recent neuroimaging studies have begun to reveal the neural correlates of circadian effects on attention and cognitive control [274]. Functional connectivity analyses demonstrate time-of-day-dependent changes in network organization that parallel behavioral performance patterns [275]. These findings suggest that circadian modulation of membrane capacitance, operating at the neuronal level, scales up to influence large-scale brain network dynamics supporting attention and executive control [276].

### 6.4. Sensory Processing and Perceptual Thresholds

Sensory systems exhibit circadian modulation at multiple levels of processing hierarchy, from peripheral receptors to cortical sensory areas [71,143]. Perceptual thresholds for detecting weak stimuli vary across the day in vision, audition, olfaction, and somatosensation, reflecting changes in both peripheral sensitivity and central processing efficiency [277,278]. Neuronal membrane capacitance contributes to sensory processing through its effects on temporal integration of sensory signals and the precision with which stimulus timing can be encoded [87].

Visual processing shows particularly well-characterized circadian rhythms, with contrast sensitivity, temporal resolution, and color discrimination all exhibiting time-of-day variations [279,280,281]. Retinal ganglion cells demonstrate circadian changes in membrane properties and firing patterns that influence visual signal transmission to central targets [282]. Cortical visual areas display circadian modulation of response properties that correlate with perceptual performance, suggesting coordinated regulation across the visual hierarchy [283].

Auditory temporal processing, critical for speech comprehension and music perception, depends on precise neuronal timing mechanisms influenced by membrane time constants [284,285]. Gap detection thresholds and temporal order judgments show circadian variation that may reflect changes in the temporal resolution of auditory neurons [286]. Recent work has demonstrated that circadian disruption impairs auditory processing in ways that cannot be explained by arousal changes alone, implicating specific alterations in neuronal biophysical properties [287].

Sensory gating, the process of filtering redundant or irrelevant sensory information, exhibits circadian regulation that influences both sensory and cognitive aspects of information processing [288,289]. Prepulse inhibition of startle responses, a measure of sensory gating, varies across circadian phase in rodents and humans [290]. Disrupted sensory gating is observed in schizophrenia and other psychiatric conditions characterized by altered circadian rhythms, suggesting mechanistic links between circadian biophysics and sensory processing abnormalities [25,291].

### 6.5. Motor Control, Coordination, and Timing

Motor performance displays pronounced circadian rhythmicity across diverse measures including strength, endurance, coordination, and timing precision [292,293,294]. These variations reflect both peripheral factors such as muscle physiology and central nervous system properties including neuronal membrane dynamics that influence motor command generation and execution [295]. The temporal coordination required for complex motor sequences depends critically on precise neuronal timing mechanisms that are sensitive to membrane capacitance [296].

Fine motor control and dexterity show time-of-day effects that correlate with core body temperature rhythms and other circadian markers [292,297,298]. Tasks requiring precise timing such as finger tapping or rapid sequential movements exhibit circadian variation in both speed and accuracy [299]. These effects likely reflect changes in motor cortex and cerebellar neuronal properties that influence movement planning and execution [300].

Motor learning and adaptation processes show circadian modulation, with different learning paradigms exhibiting distinct optimal times for acquisition or consolidation [301,302,303]. The neural plasticity mechanisms underlying motor learning depend on spike-timing-dependent processes that are influenced by membrane time constants [304]. Circadian regulation of capacitance could therefore gate motor learning by modulating the temporal windows for inducing synaptic modifications in motor circuits [34].

Balance and postural control exhibit circadian variation that has implications for fall risk and injury prevention, particularly in elderly populations [305,306]. The sensorimotor integration required for maintaining balance depends on rapid processing of vestibular, proprioceptive, and visual information within neural circuits whose temporal dynamics are shaped by membrane properties [307]. Understanding how circadian modulation of these properties affects balance control could inform strategies for reducing fall risk during vulnerable time periods [308,309].

## 7. Clinical Implications and Disease States

The disruption of circadian regulation of neuronal membrane capacitance has emerged as a common pathophysiological feature across diverse neurological and psychiatric disorders [25,80]. Understanding these connections provides insights into disease mechanisms and identifies potential therapeutic targets for chronomedicine interventions [26,79].

### 7.1. Neuropsychiatric Disorders

#### 7.1.1. Depression and Mood Disorders

Major depressive disorder (MDD) exhibits profound alterations in circadian rhythms affecting sleep–wake cycles, hormone secretion, core body temperature, and cognitive performance [310,311]. Sleep disturbances occur in up to 90% of depressed patients, with characteristic patterns including reduced REM sleep latency, increased REM density, and decreased slow-wave sleep [312,313]. While these observations establish strong correlations between circadian disruption and mood disorders, the causal relationships remain incompletely resolved. Longitudinal studies are needed to determine whether circadian dysfunction precedes, accompanies, or results from depressive episodes. Additionally, the extent to which membrane capacitance dysregulation specifically contributes to mood symptoms versus serving as a biomarker of broader circadian disruption requires targeted investigation using cell-type-specific manipulations in animal models. These sleep architecture abnormalities reflect underlying disruptions in neural circuit dynamics that depend critically on membrane biophysical properties [314].

The monoamine hypothesis of depression has been refined to incorporate circadian regulation of serotonergic, dopaminergic, and noradrenergic systems [14,315]. These neurotransmitter systems exhibit robust circadian rhythmicity in synthesis, release, and receptor expression [316]. Disruptions in circadian membrane capacitance regulation could impair the temporal coordination of monoaminergic signaling, contributing to mood dysregulation and cognitive symptoms [4,15].

Bipolar disorder presents particularly striking circadian abnormalities, with mood episodes often triggered by disruptions in sleep–wake timing or seasonal changes in photoperiod [317,318]. Genetic studies have identified associations between bipolar disorder and polymorphisms in core clock genes including CLOCK, BMAL1, and PER3 [319,320]. These findings suggest that altered circadian regulation of membrane properties may contribute to the characteristic cycling of mood states and associated changes in energy, cognition, and behavior [321].

Rapid-acting antidepressant interventions including sleep deprivation and bright light therapy demonstrate the clinical relevance of circadian timing for mood regulation [310,312,322]. These interventions likely exert therapeutic effects through acute modulation of neuronal excitability and network dynamics influenced by membrane capacitance [323]. The temporal specificity of these treatments underscores the importance of proper circadian alignment for optimal brain function [324].

#### 7.1.2. Schizophrenia, ADHD, and Autism Spectrum Disorders

Schizophrenia features prominent circadian disruptions including fragmented sleep–wake cycles, altered melatonin secretion, and disturbed social rhythms [325,326]. Patients exhibit reduced amplitude and phase delays in circadian markers that correlate with symptom severity and cognitive deficits [327]. Postmortem studies have revealed altered expression of clock genes in prefrontal cortex and other brain regions implicated in schizophrenia pathophysiology [328,329].

Sensory gating deficits characteristic of schizophrenia show circadian modulation and may reflect altered membrane properties affecting temporal filtering of sensory information [289]. Prepulse inhibition of startle, a measure of sensory gating commonly impaired in schizophrenia, exhibits time-of-day variation that is disrupted in patients [291,330]. These findings suggest that circadian dysregulation of membrane capacitance could contribute to both sensory processing abnormalities and cognitive symptoms [25].

Attention-deficit/hyperactivity disorder (ADHD) frequently co-occurs with circadian rhythm disruption, including delayed sleep phase syndrome and irregular sleep–wake patterns [331,332]. Children and adults with ADHD show altered circadian profiles of cortisol, melatonin, and core body temperature [333]. Recent evidence suggests that circadian clock genes may represent susceptibility loci for ADHD, with specific polymorphisms associated with symptom severity [334,335].

Autism spectrum disorders (ASDs) exhibit high prevalence of sleep disturbances affecting 50–80% of individuals [336,337]. Genetic studies have identified disruptions in circadian clock mechanisms in ASDs, including alterations in CLOCK gene expression and melatonin synthesis pathways [338]. The sensory hypersensitivity and altered social communication characteristic of ASDs may partly reflect disrupted circadian modulation of sensory processing and neural network dynamics [339,340].

### 7.2. Neurodegenerative Diseases

#### 7.2.1. Alzheimer’s Disease

Alzheimer’s disease (AD) presents with progressive circadian dysfunction that often precedes cognitive symptoms and worsens with disease progression [26,28]. Patients exhibit fragmented sleep–wake cycles, sundowning behavior, and dampened circadian rhythms in core body temperature and hormone secretion [341,342]. These disruptions correlate with tau pathology burden and cognitive decline, suggesting mechanistic links between circadian dysfunction and neurodegeneration [343,344].

The bidirectional relationship between sleep disruption and amyloid-beta (Aβ) pathology has been well-documented [167,345,346]. Sleep loss increases interstitial fluid Aβ levels, while Aβ accumulation disrupts sleep architecture, creating a vicious cycle accelerating disease progression [347]. Circadian regulation of glymphatic clearance mechanisms may represent a key pathway linking membrane dynamics to protein homeostasis [348,349].

Recent findings demonstrate that circadian disruption accelerates AD-like pathology in transgenic mouse models, while restoration of circadian rhythms through environmental or pharmacological interventions can ameliorate cognitive deficits [132,350]. These observations support the hypothesis that disrupted circadian regulation of membrane capacitance contributes to synaptic dysfunction and network dysregulation in AD [79,141].

The suprachiasmatic nucleus (SCN) undergoes neurodegeneration in AD, with loss of vasopressin and vasoactive intestinal peptide neurons correlating with circadian rhythm deterioration [351,352]. This central clock dysfunction propagates throughout the brain, disrupting peripheral oscillators and their outputs including membrane biophysical properties [80]. Therapeutic strategies targeting circadian restoration may therefore offer disease-modifying potential in AD [353,354].

#### 7.2.2. Parkinson’s Disease

Parkinson’s disease (PD) features prominent circadian disturbances affecting 60–90% of patients, including sleep fragmentation, excessive daytime sleepiness, and REM sleep behavior disorder [355,356]. These sleep abnormalities often precede motor symptoms by years, suggesting that circadian dysfunction represents an early pathological feature [357,358]. The loss of dopaminergic neurons in substantia nigra disrupts circadian regulation throughout the brain, given dopamine’s role in modulating clock gene expression and neuronal excitability [128,359].

Motor fluctuations in PD exhibit circadian patterns, with symptom severity varying predictably across the day [360]. These fluctuations may reflect underlying rhythms in neuronal membrane properties affecting basal ganglia circuit dynamics [361]. Recent studies have demonstrated that circadian misalignment exacerbates motor deficits in PD models, while chronotherapeutic approaches can improve motor function [362,363].

Non-motor symptoms of PD including cognitive impairment, depression, and autonomic dysfunction also show circadian variation [364,365]. The interaction between dopamine depletion and circadian disruption likely contributes to these symptoms through effects on prefrontal and limbic circuit function [366]. Understanding how altered membrane capacitance regulation impacts these circuits could inform development of time-dependent therapeutic strategies [367].

Light therapy has shown promise for treating both motor and non-motor symptoms in PD, potentially through effects on circadian alignment and neuronal excitability [368,369]. The therapeutic efficacy of properly timed interventions underscores the clinical importance of maintaining circadian regulation of membrane biophysical properties [79].

#### 7.2.3. Other Neurodegenerative Conditions

Huntington’s disease (HD) manifests circadian dysfunction early in disease course, with sleep disturbances and altered rest–activity patterns preceding cognitive and motor symptoms [370,371]. The mutant huntingtin protein disrupts clock gene expression and SCN function, leading to progressive deterioration of circadian rhythms [372,373]. Mouse models of HD demonstrate that restoring sleep–wake cycles can normalize clock gene oscillations and improve cognitive performance [374,375].

Amyotrophic lateral sclerosis (ALS) patients frequently experience sleep disruption and circadian rhythm abnormalities that impact quality of life [376,377]. While respiratory compromise contributes to sleep disturbances, evidence suggests that neurodegeneration affects circadian regulatory centers independent of ventilatory issues [378]. The role of disrupted membrane capacitance regulation in ALS pathophysiology remains an important area for future investigation [28].

Frontotemporal dementia (FTD) exhibits prominent behavioral changes including altered sleep–wake patterns and circadian rhythm disruption [379,380]. Genetic forms of FTD associated with tau and TDP-43 pathology show disrupted clock gene expression in affected brain regions [381]. The relationship between protein aggregation, circadian dysfunction, and membrane biophysical alterations represents an emerging research focus [28].

### 7.3. Epilepsy and Seizure Susceptibility

Epileptic seizures demonstrate circadian patterns of occurrence, with different seizure types showing preferred times of day [382,383]. Temporal lobe epilepsy seizures typically occur during sleep or early morning, while absence seizures predominate during waking hours [384]. These temporal patterns reflect underlying circadian modulation of neuronal excitability determined partly by membrane capacitance rhythms [16].

The perineuronal nets (PNNs) that regulate fast-spiking interneuron membrane capacitance show altered structure in epilepsy, contributing to network hyperexcitability [27,385,386]. Degradation of PNNs in epilepsy models increases membrane capacitance and reduces firing frequency of inhibitory neurons, shifting excitation–inhibition balance toward seizure generation [172]. The circadian regulation of PNN dynamics may therefore influence seizure susceptibility across the day–night cycle [22].

Antiepileptic drug efficacy shows time-of-day variation, with chronopharmacological approaches demonstrating improved seizure control and reduced side effects when dosing is optimized to circadian phase [387,388]. These findings support the concept that circadian modulation of membrane properties influences both seizure generation and pharmacological responsiveness [389,390,391,392].

Sleep deprivation powerfully increases seizure susceptibility, an effect exploited clinically for EEG activation [393,394]. The mechanisms underlying this phenomenon likely involve disrupted circadian regulation of membrane excitability and impaired homeostatic control of excitation–inhibition balance [17,229].

### 7.4. Implications for Cognitive Decline and Aging

Normal aging is accompanied by progressive dampening of circadian rhythm amplitude affecting multiple physiological and behavioral outputs [180,181]. Older adults exhibit reduced circadian modulation of sleep–wake cycles, hormone secretion, core body temperature, and cognitive performance [395,396]. These age-related changes in circadian function parallel declining neuronal membrane property regulation and may contribute to cognitive aging [77,173].

The amplitude reduction in circadian rhythms during aging reflects deterioration at multiple levels including SCN neuronal degeneration, reduced amplitude of molecular clock oscillations, and impaired coordination between central and peripheral oscillators [187,397]. These changes propagate through neural circuits to disrupt membrane capacitance regulation, affecting synaptic integration, network synchrony, and cognitive processing [79,109].

Interventions that strengthen circadian rhythms in aging populations show promising effects on cognitive function [398,399]. Increased light exposure, regular exercise timing, and scheduled social activities can enhance circadian amplitude and improve sleep quality, attention, and memory performance [354,400]. These behavioral approaches likely exert benefits through restoration of proper membrane biophysical regulation [132].

The concept of “cognitive aging” as distinct from neurodegenerative disease is increasingly challenged by evidence that age-related cognitive decline and dementia exist on a continuum [401,402]. Circadian dysfunction may accelerate progression along this continuum by impairing neuronal function and promoting protein aggregation through disrupted proteostasis [28,349]. Maintaining robust circadian regulation of membrane capacitance throughout the lifespan may therefore represent an important strategy for preserving cognitive health [79,119]. The major neurological and psychiatric disorders associated with disrupted circadian regulation of neuronal membrane capacitance, their molecular mechanisms, and therapeutic implications are summarized in Table 3.

## 8. Translational Perspectives and Chronomedicine

The integration of circadian biophysics with clinical practice represents a frontier in precision medicine, offering opportunities to optimize therapeutic interventions by aligning treatments with the temporal organization of biological systems [403,404]. Understanding how circadian regulation of membrane capacitance influences neuronal function provides a mechanistic framework for developing time-based therapeutic strategies [36].

### 8.1. Chronotherapeutic Strategies

Chronotherapy, the practice of timing therapeutic interventions to circadian phase, has demonstrated efficacy across diverse medical conditions including cardiovascular disease, cancer, and psychiatric disorders [405,406]. The rationale for chronotherapy rests on the principle that drug pharmacokinetics and pharmacodynamics vary across the circadian cycle due to rhythmic changes in absorption, distribution, metabolism, and target tissue sensitivity [407,408]. These temporal variations can result in up to ten-fold differences in treatment efficacy and toxicity depending on administration time [409,410].

In psychiatric care, chronotherapeutic approaches show particular promise for mood disorders where circadian disruption represents a core pathophysiological feature [14,310]. Sleep deprivation therapy produces rapid antidepressant effects in 40–60% of patients with major depression, with response rates higher than many pharmacological interventions [411,412]. The therapeutic mechanism likely involves acute modulation of neuronal excitability and network dynamics through effects on membrane properties and neurotransmitter systems [413]. However, relapse rates are high unless sleep deprivation is combined with other interventions such as light therapy or sleep phase advance [414,415].

Bright light therapy represents another well-established chronotherapeutic modality for mood disorders, seasonal affective disorder, and circadian rhythm sleep disorders [322,416]. Light exposure at specific circadian phases shifts the timing of the SCN pacemaker and its downstream outputs, potentially restoring proper alignment between internal clocks and external demands [24,417]. Recent evidence suggests that light therapy exerts effects beyond simple phase shifting, including direct modulation of brain regions involved in mood regulation and alertness [418,419].

Pharmacological chronotherapy requires careful consideration of drug-specific pharmacological properties and their interaction with circadian physiology [409,420]. Antihypertensive medications show administration-time-dependent efficacy, with evening dosing of certain agents producing superior blood pressure control and reduced cardiovascular event risk compared to morning administration [421,422]. Similar principles apply to neuropsychiatric medications, where the timing of antidepressant, antipsychotic, or mood stabilizer administration may influence both therapeutic efficacy and side effect profiles [423,424].

The development of time-release formulations and chronopharmaceutical drug delivery systems enables more precise temporal targeting of therapeutic interventions [425,426]. These approaches can optimize drug exposure patterns to match circadian rhythms in target tissue sensitivity or disease activity [406]. For neurological applications, drug delivery systems that account for blood–brain barrier permeability rhythms could enhance CNS drug penetration while minimizing systemic exposure [427,428].

### 8.2. Personalized Medicine and Chronotype-Based Interventions

Individual differences in circadian timing, reflected in chronotype preference ranging from extreme morningness to extreme eveningness, have profound implications for personalized medicine [256,429,430]. Chronotype is determined by both genetic factors, including polymorphisms in core clock genes, and environmental influences such as light exposure patterns and social schedules [431,432]. The mismatch between endogenous circadian phase and societal demands, termed social jetlag, affects a substantial proportion of the population and associates with increased disease risk [433,434].

Personalized chronotherapy requires assessment of individual circadian phase to optimize intervention timing [429,435,436]. Phase markers including dim light melatonin onset (DLMO), core body temperature minimum, and actigraphy-derived sleep parameters can guide treatment scheduling [437,438]. Advanced computational approaches using machine learning algorithms to predict optimal treatment windows based on individual circadian profiles represent an emerging frontier in precision chronomedicine [439,440].

Genetic chronotyping through analysis of clock gene polymorphisms may eventually enable prediction of individual circadian characteristics and treatment responsiveness [441,442,443]. Variants in genes including PER3, CLOCK, and BMAL1 influence not only circadian phase preference but also cognitive performance patterns, mood disorder susceptibility, and drug metabolism [444,445]. Integration of genetic information with behavioral and physiological circadian assessments could refine personalized treatment algorithms [429,441,446,447].

The synchrony effect, whereby cognitive performance is enhanced when task demands align with individual chronotype preference, has important implications for scheduling cognitive rehabilitation, educational interventions, and workplace activities [249,250]. Older adults show particularly strong synchrony effects for executive function tasks, with performance deficits when tested at non-optimal circadian phases [247,269]. Recognition of these individual differences can inform personalized strategies for optimizing cognitive function across the lifespan [11].

### 8.3. Technological and Socio-Environmental Approaches

Technological advances in wearable devices and smartphone applications enable continuous monitoring of circadian rhythms through measures including activity patterns, sleep–wake timing, heart rate variability, and skin temperature [448,449]. These consumer-grade technologies democratize access to circadian assessment and provide data for personalized intervention design [450,451]. Integration of multimodal sensor data through artificial intelligence approaches can extract circadian phase information with accuracy approaching laboratory-based methods [452,453].

Environmental design interventions represent non-pharmacological strategies for supporting circadian health [454,455]. Architectural lighting design incorporating tunable LED systems that mimic natural daylight patterns can enhance circadian entrainment in settings including healthcare facilities, schools, and workplaces [456,457]. Dynamic lighting protocols that provide bright, blue-enriched light during the day and dim, warm light in the evening support proper circadian alignment and improve sleep quality and daytime alertness [458,459].

Sleep hygiene education and behavioral interventions targeting circadian alignment show efficacy for improving sleep disorders and mental health outcomes [460,461]. Cognitive behavioral therapy for insomnia (CBT-I) incorporates circadian principles including consistent sleep–wake scheduling and strategic light exposure [462,463]. Extending these approaches to explicitly target circadian regulation of membrane biophysical properties through activity timing and environmental manipulations represents a logical next step [324].

Social timing interventions acknowledge that human circadian rhythms are entrained not only by light but also by social cues including meal timing, exercise, and social interactions [75,464]. Structured daily routines that provide consistent timing signals can strengthen circadian rhythms and improve health outcomes, particularly in populations vulnerable to circadian disruption such as shift workers, older adults, and individuals with psychiatric conditions [465,466].

### 8.4. Challenges and Future Directions

Despite promising developments, significant challenges impede translation of circadian biophysics research into clinical applications [35,36]. The complexity of circadian systems, with their hierarchical organization and tissue-specific outputs, complicates prediction of intervention effects [37]. Individual variability in circadian parameters and treatment responsiveness necessitates personalized approaches that may not be feasible in all clinical settings [24,467].

Several key questions remain unresolved and represent priorities for future investigation:

1. Causal mechanisms: While correlations between Cm oscillations and behavioral/cognitive rhythms are established, causal links require systematic testing using optogenetic or chemogenetic manipulation of specific molecular pathways in defined neuronal populations.
2. Cell-type specificity: The differential regulation of Cm across neuronal subtypes (e.g., excitatory vs. inhibitory, cortical vs. subcortical) and the functional consequences of this heterogeneity for circuit computation remain incompletely characterized.
3. Human translation: Nearly all mechanistic data derive from rodent studies. Validation of key findings in human tissue (e.g., surgically resected brain samples, induced pluripotent stem cell-derived neurons) is essential for clinical translation.
4. Measurement challenges: Development of non-invasive biomarkers reflecting neuronal Cm rhythms would enable longitudinal tracking in patients and assessment of therapeutic interventions.
5. Specificity of interventions: Current chronotherapeutic approaches (light therapy, sleep manipulation) affect multiple systems simultaneously. Development of targeted interventions selectively modulating Cm regulation would enable mechanistic testing and potentially reduce side effects.

Standardization of circadian assessment methods represents an important priority for chronomedicine implementation [438,468]. While laboratory-based protocols using constant routine or forced desynchrony paradigms provide gold-standard circadian phase estimation, their impracticality for routine clinical use limits applicability [469,470]. Development and validation of practical circadian biomarkers that can be obtained in ambulatory settings would facilitate personalized chronotherapy [440,471].

The mechanisms linking circadian membrane capacitance regulation to clinical phenotypes require further elucidation through translational research bridging cellular biophysics and systems-level function [28]. Advanced electrophysiological techniques enabling longitudinal membrane property measurements in behaving animals could reveal dynamic relationships between biophysical rhythms and behavior [120,472]. Optogenetic and chemogenetic approaches for manipulating clock gene expression in specific neuronal populations offer tools for establishing causal relationships [473,474].

Ethical considerations arise regarding chronotype-based scheduling in educational and occupational settings [475]. While accommodating individual circadian preferences could improve performance and wellbeing, practical constraints and equity concerns must be addressed [433,476]. Policy initiatives supporting flexible scheduling and awareness of chronotype diversity represent important steps toward circadian-informed social structures [477].

The development of novel pharmacological agents specifically targeting circadian clock mechanisms represents an emerging therapeutic frontier [478,479]. Small-molecule modulators of clock proteins, including CRY stabilizers and REV-ERB agonists, show promise in preclinical models for metabolic disease, cancer, and neuropsychiatric conditions [480,481]. Extending these approaches to target membrane biophysical regulation through clock-controlled pathways could yield innovative treatments for neurological disorders [54].

Integration of circadian principles into clinical trial design would enhance detection of treatment effects and reduce variability [410,482]. Accounting for circadian phase at assessment time points, incorporating chronotype as a stratification variable, and timing interventions to optimal circadian phases could improve trial outcomes [406,409]. Regulatory pathways for chronotherapeutic claims need development to facilitate clinical translation while ensuring evidence standards [483,484].

## 9. Conclusions

The circadian regulation of neuronal membrane capacitance represents a fundamental organizing principle linking molecular timekeeping to neural computation, behavior, and health. The evidence synthesized in this review establishes that membrane capacitance is not a static biophysical parameter but instead exhibits robust daily oscillations that profoundly influence neuronal information processing across multiple temporal and spatial scales. These oscillations emerge from coordinated molecular mechanisms including transcriptional control of membrane-associated genes, lipid metabolic rhythms, ion channel trafficking dynamics, and glial-mediated extracellular matrix remodeling.

At the single-neuron level, circadian modulation of membrane capacitance directly alters the membrane time constant, thereby shifting neurons between temporal integration and coincidence detection computational modes across the day–night cycle. These changes in temporal filtering properties propagate through neural circuits to influence synaptic plasticity, network oscillations, and ultimately cognitive performance in domains including memory consolidation, working memory, attention, sensory processing, and motor control. The functional significance of capacitance rhythms is underscored by observations that disruptions in these biophysical oscillations are consistently observed across diverse neuropsychiatric and neurodegenerative disorders.

The clinical implications of disrupted circadian capacitance regulation extend from mood disorders and schizophrenia to Alzheimer’s disease, Parkinson’s disease, and epilepsy, suggesting that restoration of healthy biophysical rhythms may represent a therapeutic strategy with broad applicability. The integration of circadian biophysics with chronomedicine offers promising avenues for optimizing treatment efficacy through chronotherapeutic approaches that align interventions with intrinsic biological timing. Personalized medicine strategies incorporating individual chronotype assessment and genetic profiling could further refine temporal targeting of therapies while minimizing adverse effects.

Several key priorities emerge for future research. First, mechanistic studies are needed to fully elucidate the molecular pathways linking core clock components to membrane capacitance regulation, including the specific roles of lipid metabolic enzymes, ion channel trafficking machinery, and glial factors. Second, translational investigations must establish causal relationships between altered capacitance rhythms and clinical phenotypes through longitudinal studies combining electrophysiological measurements with behavioral and cognitive assessments. Third, technological development of practical biomarkers enabling routine assessment of circadian phase and membrane biophysical status would facilitate implementation of personalized chronotherapy in clinical settings.

The development of novel therapeutic strategies specifically targeting circadian regulation of membrane properties represents an important frontier. Pharmacological agents modulating clock proteins, lipid metabolic pathways, or ion channel trafficking could restore healthy capacitance rhythms in disease states. Environmental interventions including optimized lighting protocols and structured behavioral scheduling offer complementary non-pharmacological approaches. The integration of these strategies within comprehensive treatment plans accounting for individual circadian characteristics holds promise for improving outcomes across neurological and psychiatric conditions.

From a broader perspective, understanding circadian regulation of membrane capacitance illuminates fundamental principles of neural circuit organization and temporal coordination. The recognition that even passive biophysical properties undergo dynamic regulation challenges traditional views of neuronal function and highlights the pervasive influence of time-of-day on brain operations. This temporal dimension must be incorporated into computational models of neural information processing, experimental designs in neuroscience research, and clinical assessment protocols.

The integrative framework presented in this review provides a foundation for future experimental and computational investigations of circadian membrane biophysics. Experimentally, this framework suggests several priorities: first, cell-type-specific interventions targeting lipid biosynthetic enzymes, ion channel trafficking proteins, or perineuronal net components, combined with longitudinal membrane capacitance measurements across circadian phases; second, simultaneous assessment of molecular clock gene expression, electrophysiological properties, and behavioral performance to establish temporal correlations across organizational levels; and third, validation of rodent-derived findings in human tissue, including surgically resected samples or induced pluripotent stem cell-derived neurons. Computationally, incorporation of time-dependent capacitance parameters into biophysically realistic neuron models would enable systematic testing of hypotheses regarding how circadian modulation of membrane properties influences temporal integration, spike timing precision, and network synchronization. Multi-scale modeling approaches linking molecular oscillators to cellular biophysics and circuit-level computation could identify critical regulatory nodes where interventions might restore healthy membrane dynamics in disease states, thereby informing rational chronotherapeutic design.

The convergence of circadian biology, cellular biophysics, and systems neuroscience exemplified by research on membrane capacitance rhythms illustrates the power of interdisciplinary approaches to advance understanding of brain function in health and disease. Continued integration across these domains, enabled by technological advances in electrophysiology, imaging, genomics, and computational modeling, will deepen our appreciation of how molecular clocks orchestrate the temporal structure of neural computation and behavior.

Ultimately, the circadian regulation of neuronal membrane capacitance serves as a vivid example of how biological rhythms are not mere epiphenomena but instead represent fundamental organizing principles essential for optimal function. By rhythmically modulating the biophysical substrate of neural computation, circadian clocks ensure that brain circuits operate with temporal coordination appropriate for the behavioral and environmental demands that vary predictably across the 24 h day. Disruptions to these rhythms, whether through genetic, environmental, or pathological factors, compromise this temporal organization with far-reaching consequences for cognition, behavior, and health. Recognizing and restoring proper circadian regulation of membrane biophysics therefore represents both a scientific imperative and a clinical opportunity to improve human wellbeing.

## Figures and Tables

**Table 1:** Ion channel trafficking and capacitance modulation across circadian cycles.

Channel Type	Circadian Regulation Mechanism	Time-of-Day Variation	Functional Consequence
**Voltage-gated K** ^+^ **channels (Kv2.1)**	Activity-dependent redistribution modulated by circadian factors Rhythmic phosphorylation states Changes in clustering patterns Post-translational modifications (phosphorylation, ubiquitination, palmitoylation)	Clustering patterns vary with time of day Phosphorylation states show circadian rhythmicity	Altered spatial distribution affects membrane electrical properties Changes in membrane capacitance through modified channel density Influences action potential repolarization kinetics Modulates neuronal excitability across circadian cycle
**Voltage-gated Na** ^+^ **channels**	Circadian variations in axon initial segment (AIS) density Rhythmic trafficking via Rab proteins and SNARE-mediated membrane fusion Transcriptional oscillations of channel genes Clock-regulated kinase activities affecting trafficking rates	Density variations at axon initial segment correlate with circadian time Peak expression timing affects action potential initiation threshold	Changes in neuronal excitability Altered action potential initiation dynamics Modified firing rate capabilities Influences membrane charging rate and integration properties Affects membrane time constant (τm)
**Voltage-gated Ca^2^** ^+^ **channels**	Complex trafficking regulation via auxiliary subunits Circadian modulation of auxiliary subunits influences channel stability at membrane Rhythmic expression and activity of Rab proteins coordinating vesicular transport	Time-of-day-dependent variations in channel density and distribution Auxiliary subunit composition varies across circadian phases	Modified gating kinetics and voltage dependence Time-of-day-specific channel phenotypes Altered Ca^2+^ influx affects synaptic transmission and plasticity Influences membrane capacitance through changes in channel surface expression Affects integration of synaptic inputs during memory consolidation
**General voltage-gated channels**	Continuous cycles of endocytosis and reinsertion via circadian-regulated pathways SNARE proteins displaying circadian modulation control membrane fusion during channel insertion Circadian regulation of channel auxiliary subunits	Density and spatial distribution show time-of-day-dependent variations	Influences both membrane electrical properties and action potential dynamics Fine-tunes membrane responses for different behavioral contexts Complements capacitance changes to optimize neuronal computation Affects temporal precision of neuronal synchronization during sleep oscillations Modulates temporal integration windows for detecting coincident synaptic inputs during memory reactivation

Abbreviations: AIS, axon initial segment; τm, membrane time constant.

**Table 2:** Mechanisms regulating circadian oscillations of neuronal membrane capacitance.

Regulatory Level	Key Mechanisms	Molecular Components	Functional Outcome
**Transcriptional regulation**	CLOCK-BMAL1 heterodimers bind E-box elements in promoters of membrane-associated genes Secondary feedback loops control lipid biosynthetic pathways Rhythmic expression of ion channel genes	CLOCK, BMAL1 (core clock transcription factors) REV-ERBα, RORα (nuclear receptors) Per2 (clock gene) Voltage-gated Na^+^, K^+^, Ca^2+^ channel genes	Time-of-day-dependent modulation of membrane protein expression Coordination of membrane remodeling with cellular energy status Cm oscillations with 15–20% amplitude from baseline Cell-type-specific rhythmicity patterns
**Lipid metabolism**	Circadian oscillations in phospholipid biosynthesis Rhythmic activity of lipid synthesis and degradation enzymes Sphingolipid metabolism in glial-neuronal interactions Coordinated balance of anabolic/catabolic pathways	Fatty acid synthase Acetyl-CoA carboxylase CDP-choline pathway enzymes Phospholipases Glucocerebrosidase (gba1b) SREBP (lipid metabolism regulator)	Dynamic membrane remodeling alters bilayer composition and thickness ↑ Phosphatidylcholine, phosphatidylethanolamine at specific phases Changes in membrane dielectric properties → Bidirectional modulation of Cm depending on phase Altered membrane surface area affects total capacitance
**Ion channel trafficking and localization**	Rhythmic endocytosis/exocytosis of channels Activity-dependent redistribution modulated by circadian factors Post-translational modifications affecting trafficking rates Dynamic regulation of channel auxiliary subunits	Rab proteins (GTPases coordinating vesicular transport) SNARE proteins (membrane fusion) Kv2.1, Kv channels (voltage-gated K^+^) Na^+^ channels Ca^2+^ channels and auxiliary subunits HCN channels	Time-of-day-dependent variations in channel density Altered spatial distribution affects membrane electrical properties ↑/↓ Channel surface expression modulates Cm Modified gating kinetics and voltage dependence Changes in neuronal excitability and action potential dynamics
**Glial modulation**	Oligodendrocyte circadian rhythms affect myelin dynamics Astrocytic morphological remodeling varies with circadian time Rhythmic assembly/disassembly of perineuronal nets (PNNs) Control of extracellular space geometry and ionic composition	Matrix metalloproteinases (MMP, PNN remodeling) Chondroitin sulfate proteoglycans Hyaluronan and link proteins Oligodendrocyte metabolic enzymes Astrocytic process regulatory factors	Changes in myelin thickness alter effective Cm (↓ specific capacitance) Myelin geometry optimization affects conduction velocity Astrocytic ensheathment influences local electrical field properties PNN density modulation alters neuronal microenvironment Phase-specific adjustments in membrane time constant (τm) Glia-driven rhythmic modulation of neuronal Cm and τm

Abbreviations: Cm = membrane capacitance; τm = membrane time constant. Arrows (↑/↓) indicate increases or decreases in capacitance.

**Table 3:** Disorders Associated with Disrupted Circadian Regulation of Membrane Capacitance.

Disorder	Type of Capacitance Disruption	Underlying Mechanisms	Behavioral/Cognitive Consequence	Therapeutic Implication
**Major Depressive Disorder (MDD)**	Disrupted circadian rhythms affecting sleep–wake cycles Altered neural circuit dynamics dependent on membrane biophysical properties	Disrupted circadian regulation of serotonergic, dopaminergic, and noradrenergic systems Impaired temporal coordination of monoaminergic signaling Disrupted circadian membrane capacitance regulation	Sleep disturbances (90% of patients) Reduced REM sleep latency, increased REM density Decreased slow-wave sleep Mood dysregulation and cognitive symptoms	Sleep deprivation therapy Bright light therapy Temporal specificity of treatments for circadian alignment Acute modulation of neuronal excitability
**Bipolar Disorder**	Altered circadian regulation of membrane properties Disruptions in sleep–wake timing	Polymorphisms in core clock genes (CLOCK, BMAL1, PER3) Altered circadian regulation contributing to mood cycling	Mood episodes triggered by sleep–wake disruptions or seasonal photoperiod changes Cycling of mood states Changes in energy, cognition, and behavior	Circadian alignment interventions Bright light therapy Sleep–wake schedule stabilization
**Schizophrenia**	Reduced amplitude circadian rhythms Phase delays in circadian markers Altered membrane properties affecting temporal filtering	Altered expression of clock genes in prefrontal cortex Circadian dysregulation of membrane capacitance affecting sensory gating	Fragmented sleep–wake cycles Altered melatonin secretion Disturbed social rhythms Sensory gating deficits (disrupted prepulse inhibition) Cognitive deficits correlating with circadian amplitude reduction Sensory processing abnormalities	Restoration of circadian rhythms Sleep–wake stabilization Interventions targeting sensory gating improvement
**Attention-Deficit/Hyperactivity Disorder (ADHD)**	Circadian rhythm disruption Irregular sleep–wake patterns	Circadian clock genes as susceptibility loci Specific polymorphisms associated with symptom severity Altered circadian profiles of cortisol, melatonin, core body temperature	Delayed sleep phase syndrome Irregular sleep–wake patterns Symptom severity variation with circadian disruption	Circadian rhythm stabilization Sleep schedule optimization Chronotherapeutic approaches
**Autism Spectrum Disorders (ASDs)**	Disrupted circadian modulation of sensory processing and neural network dynamics	Disruptions in circadian clock mechanisms Alterations in CLOCK gene expression Disrupted melatonin synthesis pathways	Sleep disturbances (50–80% of individuals) Sensory hypersensitivity Altered social communication	Melatonin supplementation Sleep hygiene interventions Circadian rhythm stabilization
**Alzheimer’s Disease (AD)**	Progressive circadian dysfunction Disrupted circadian regulation of membrane capacitance Loss of rhythmicity	SCN neurodegeneration (loss of vasopressin and VIP neurons) Central clock dysfunction propagating to peripheral oscillators Disrupted circadian regulation of glymphatic clearance Disrupted membrane biophysical properties output	Fragmented sleep–wake cycles Sundowning behavior Dampened circadian rhythms in temperature and hormone secretion Impaired memory consolidation Synaptic dysfunction and network dysregulation •Bidirectional relationship: sleep disruption increases Aβ, Aβ disrupts sleep	Environmental interventions for circadian restoration Pharmacological interventions for rhythm restoration Light therapy Circadian restoration as disease-modifying strategy
**Parkinson’s Disease (PD)**	Circadian disturbances affecting 60–90% of patients Underlying rhythms in neuronal membrane properties affecting basal ganglia circuits	Loss of dopaminergic neurons disrupting circadian regulation Dopamine’s role in modulating clock gene expression and neuronal excitability Interaction between dopamine depletion and circadian disruption	Sleep fragmentation Excessive daytime sleepiness REM sleep behavior disorder Motor fluctuations with circadian patterns Non-motor symptoms: cognitive impairment, depression, autonomic dysfunction with circadian variation	Light therapy for motor and non-motor symptoms Chronotherapeutic approaches improving motor function Time-dependent therapeutic strategies Properly timed interventions for circadian alignment
**Huntington’s Disease (HD)**	Circadian dysfunction early in disease Progressive deterioration of circadian rhythms Loss of rhythmicity	Mutant huntingtin protein disrupts clock gene expression Disrupted SCN function	Sleep disturbances and altered rest–activity patterns preceding cognitive/motor symptoms Progressive cognitive and motor decline	Restoration of sleep–wake cycles normalizes clock gene oscillations Improved cognitive performance with circadian restoration
**Amyotrophic Lateral Sclerosis (ALS)**	Circadian rhythm abnormalities Disrupted membrane capacitance regulation (area for future investigation)	Neurodegeneration affects circadian regulatory centers	Sleep disruption Quality of life impact	Sleep disorder management Circadian rhythm stabilization
**Frontotemporal Dementia (FTD)**	Circadian rhythm disruption Membrane biophysical alterations	Tau and TDP-43 pathology associated with disrupted clock gene expression Protein aggregation linked to circadian dysfunction	Altered sleep–wake patterns Behavioral changes	Circadian restoration approaches Sleep–wake stabilization
**Epilepsy**	Circadian modulation of neuronal excitability via membrane capacitance rhythms Altered PNN structure affecting membrane capacitance	Degradation of perineuronal nets (PNNs) increases membrane capacitance Reduced firing frequency of inhibitory neurons Circadian regulation of PNN dynamics influences seizure susceptibility	Circadian patterns of seizure occurrence (temporal lobe seizures during sleep/early morning; absence seizures during waking) Network hyperexcitability Shifted excitation–inhibition balance Sleep deprivation increases seizure susceptibility	Chronopharmacological approaches Optimized drug dosing to circadian phase Improved seizure control with circadian-aligned treatment Sleep schedule optimization
**Normal Aging and Cognitive Decline**	Progressive dampening of circadian rhythm amplitude Reduced circadian modulation of membrane property regulation Loss of rhythmic amplitude	SCN neuronal degeneration Reduced amplitude of molecular clock oscillations Impaired coordination between central and peripheral oscillators Disrupted proteostasis through impaired circadian regulation	Reduced circadian modulation of sleep–wake cycles, hormone secretion, temperature, cognitive performance Impaired synaptic integration and network synchrony Progressive decline in cognitive processing speed and network synchrony Accelerated progression toward dementia continuum Protein aggregation	Increased light exposure Regular exercise timing Scheduled social activities Circadian amplitude enhancement Behavioral approaches for membrane biophysical regulation restoration Maintaining robust circadian regulation throughout lifespan

Abbreviations: PNN = perineuronal nets; SCN = suprachiasmatic nucleus; VIP = vasoactive intestinal peptide; Aβ = amyloid-beta.

## Data Availability

No new data were created or analyzed in this study. Data sharing is not applicable to this article.
